# A Woman's Reason

**Published:** 1897-12-04

**Authors:** 


### A WOMAN'S REASON.

We must not pry too much into motives if only the result is good, and if we can gain sanitary benefits from ladies' love of dress by all means let us do so. The Jersey City Board of Health has passed an ordinance making expectoration on the floors or platforms of public conveyances of any kind punishable by the imposition of a 10 dols. fine. This is a purely sanitary proceeding, undertaken with the object of protecting the public health, and as such deserves the support of all good citizens. It is interesting to learn from the papers, however, that, though the ordinance was recommended by the health inspector, "its passage was urged by many women, who complained that the filthy habit had frequently caused the destruction of their dresses." A woman's reason may sometimes have but small relation with the point at issue; but who shall grumble so long as it serves its purpose?

